# Comparative efficacy of Pirfenidone and Meloxicam on early radiotherapy-induced anal sphincter dysfunction in rats

**DOI:** 10.3389/fphar.2025.1441011

**Published:** 2025-03-27

**Authors:** Dragoș Viorel Scripcariu, Cezar Cătălin Caratașu, Mitică Ciorpac, Teodora Alexa-Stratulat, Andrei Szilagyi, Cristian Răzvan Buga, Bogdan Ionuţ Dobrovăț, Lucian Eva, Andrei Daniel Timofte, Ludmila Lozneanu, Irina-Draga Căruntu, Radu Iliescu, Bogdan Ionel Tamba

**Affiliations:** ^1^ Department of Surgery, “Grigore T. Popa” University of Medicine and Pharmacy, Iasi, Romania; ^2^ Advanced Center for Research and Development in Experimental Medicine “Prof. Ostin C. Mungiu” (CEMEX), “Grigore T. Popa” University of Medicine and Pharmacy, Iasi, Romania; ^3^ Department of Medical Oncology and Radiotherapy, “Grigore T. Popa” University of Medicine and Pharmacy, Iasi, Romania; ^4^ Stereotactic Radiosurgery Laboratory, Clinical Emergency Hospital “Prof. Dr. Nicolae Oblu”, Iasi, Romania; ^5^ Department of Morphofunctional Sciences 1, “Grigore T. Popa” University of Medicine and Pharmacy, Iasi, Romania

**Keywords:** TGF-β inhibition, radiation induced-anal sphincter dysfunction, antifibrotic therapy, pirfenidone in radiotherapy-induced anal sphincter dysfunction, irradiated anal sphincter remodelling

## Abstract

**Background:**

Radiation therapy, integral to pelvic tumor management, impacts over half of all cancer patients and may lead to anal sphincter dysfunction due to inflammatory responses and chronic fibrotic remodeling in irradiated tissues. To address this, a targeted animal model has been developed to investigate early post-radiotherapy anal toxicity and evaluates the efficacy of anti-fibrotic and anti-inflammatory agents, Pirfenidone and Meloxicam, as potential treatments against radiotherapy-induced sphincter dysfunction.

**Methods:**

Thirty male Sprague Dawley rats received a 30Gy dose via stereotactic body radiotherapy targeting the anal canal and sphincter. For 28 days, anal sphincter functionality was assessed using anorectal manometry, involving electrostimulation of the perianal area. Histological evaluations were conducted to qualitatively and quantitatively analyze morphological changes and measure sphincter thickness, providing insights into post-radiation structural integrity.

**Results:**

Irradiated animals exhibited signs of perianal inflammation, without severe complications such as strictures or perforations. Functional assessments showed altered sphincter contractility, with irradiated animals initially displaying increased contraction parameters, which subsequently declined to levels below baseline measurements. The groups treated with Pirfenidone, alone and in combination with Meloxicam exhibited significant improvements in sphincter contractility and showed a notable mitigation in external anal sphincter thickness, concomitant with reduction in collagen deposition and preservation of muscular tissue, compared with untreated irradiated animals.

**Conclusion:**

This study demonstrates that Pirfenidone, either as monotherapy or in combination with Meloxicam, mitigates radiation-induced fibrotic remodeling and preserves anal sphincter function. However, the combination therapy did not provide an additive benefit over Pirfenidone alone. These findings highlight Pirfenidone as a promising therapeutic strategy for managing post-radiotherapy sphincter dysfunction. Further research is needed to elucidate the underlying molecular mechanisms and optimize antifibrotic and myoprotective interventions for clinical application in cancer survivors.

## 1 Introduction

In the last few decades, radiation therapy has become an essential tool in the management of solid tumours both in the curative and in the palliative setting, with more than 50% of cancer patients receiving radiotherapy at some point throughout their cancer journey (Baskar et al., 2012). Furthermore, recent advancements in the accessibility and clinical implementation of radiation therapy have notably increased the survival rates among patients undergoing treatment for breast, cervical, genito-urinary, and rectal cancers (Bryant et al., 2017; Roy et al., 2017). However, exposure to ionized radiation can lead to numerous adverse effects, especially in patients subjected to pelvic radiotherapy. According to Olopade et al. (2005), nearly nine out of ten patients undergoing pelvic radiotherapy experience persistent alterations in bowel habits, with half of them noting a considerable decline in their overall quality of life.

Pelvic radiotherapy induces collateral damage on adjacent tissues, encompassing muscles, blood vessels, and nerves, thereby engendering anorectal dysfunction through a multifaceted interplay of tissue injury, fibrotic processes, and compromised blood perfusion (Lundby et al., 2005). This cascade promotes collagen deposition and fibrosis, deleteriously affecting muscle elasticity and contractility (Citrin and Mitchell, 2017). Faecal incontinence arises as a consequence of radiotherapy-induced tissue damage and fibrosis within the pelvic region, including muscular dysfunction and altered rectal compliance, which challenges both patients and healthcare providers (Bruheim et al., 2010). The incidence of faecal incontinence post-radiotherapy is influenced by factors such as cancer subtype, radiation dosage, patient characteristics, and the aggressiveness of the therapeutic regimen, afflicting 5%–15% of individuals subjected to pelvic radiotherapy (Yeoh et al., 2012).

Previous investigations have provided foundational insights into the complex consequences of pelvic radiotherapy on the anorectal area, yet the exact effects on the anal canal and rectum are still not fully understood. This gap in knowledge has motivated the development of a targeted animal model to investigate the anorectal dysfunction post-radiotherapy. Initial studies, such as Hubmann’s research (Hubmann, 1981), assessed the tolerance rats’ rectum to X-ray irradiation, evaluating hemorrhagic proctitis and rectal obstruction. Histological findings indicated significant submucosal fibrosis and rectal stiffening, underscoring the challenge of optimizing tumour treatment efficacy while minimizing tissue damage. Further exploration into the effects of age on radiosensitivity and rectal complications in rats revealed that age did not significantly influence the incidence of rectal complications, suggesting that late complications may stem from early epithelial damage (van den Aardweg et al., 2003). Henke’s study Henke et al. (1996) on stereotactic radiosurgery for prostate tumors in Copenhagen rats aimed to assess the method’s efficacy in tumour control and observed histological changes post-treatment, indicating reduced cell proliferation and increased connective tissue. This suggested the potential of radiosurgery for precise tumour management with minimal impact on surrounding tissues. Additionally, Hrycushko’s study Hrycushko et al. (2017) on the radioprotective effects of local hypothermia during stereotactic radiation therapy for prostate cancer in Sprague-Dawley rats demonstrated an increased resistance to radiation-induced damage, highlighting local hypothermia as a promising strategy to mitigate radiation side effects in prostate cancer treatments. These studies collectively underscore the critical need for a specialized animal model to elucidate the detailed impacts of radiotherapy on anorectal dysfunction. The creation of such a model represents a significant advancement in the field, providing a focused framework for the exploration of therapeutic interventions against radiation-induced anorectal dysfunction and marking a pivotal step in the development of targeted treatment strategies.

The mitigation of radiation-induced fibrosis and sphincteric insufficiency through judicious therapeutic interventions is critical in preserving post-radiotherapy quality of life and the overall functional integrity of individuals under treatment. It is well-acknowledged that conventional therapeutic modalities have demonstrated limitations (Peeters et al., 2006). Consequently, the exploration of innovative therapeutic pathways specifically designed to target the fibrotic processes that underlie anorectal dysfunction has become necessary. In this regard, targeted anti-fibrotic therapies stand out as promising agents of therapeutic intervention. These therapeutic approaches, characterized by their capacity to selectively impede the pivotal pathways implicated in aberrant tissue remodelling and deposition of fibrous tissue, aim to not only interrupt but also potentially reverse the fibrotic sequelae of radiotherapy (Martin et al., 2000).

Considering these multifaceted challenges, the present study aimed to: (*i*) develop a rat model of anal sphincter impairment secondary to targeted stereotactic body radiation therapy (SBRT) and (*ii*) evaluate an innovative therapeutic strategy for mitigating post-radiotherapy anal sphincter dysfunction. First, we used a gamma knife for targeted delivery of ionizing radiation to the anal canal and anal sphincter. This approach ensured that neighbouring structures were not affected, thus allowing for specific evaluation of radiation-induced internal and external anal sphincter dysfunction. Furthermore we evaluated the efficacy of Pirfenidone, a novel anti-fibrotic agent, in comparison with Meloxicam, a non-steroidal anti-inflammatory drug (NSAID), in preventing radiation-induced structural and functional anal injury. All the experiments were carried out simultaneously, but for an easier understanding and a clearer presentation of the study’s objectives, the methodology and results will be presented separately.

## 2 Materials and methods

### 2.1 Animals

The study was performed on 30 Sprague Dawley male rats (36-week-old), with weights between 300 g and 400 g. The rats were housed in a controlled environment maintained at a temperature of 21°C–23°C with regulated humidity, following a 12-h light/dark cycle. Throughout the study, the animals had unrestricted access to food and water, and an acclimatization period of 2 weeks was provided prior to commencing the experimental procedures. The ethical aspects of the research were adhered to the European Directive 2010/63/EU on the Protection of Animals Used for Scientific Purposes and were reviewed and approved by the University Research Ethics Committee (no. 130/1.12.2021) and the National Sanitary Veterinary and Food Authority (no. 52/25.03.2022). The animals underwent daily monitoring, including maintenance tasks such as measuring water and food intake, monitoring behaviour, general appearance, and stool consistency. Additionally, an exhaustive weekly examination encompassed 16 animal welfare indicators was conducted.

The primary objective of this study was to evaluate the effects of ionizing radiation on the anal sphincter through a comprehensive analysis of general, functional, and histological parameters. The two groups comprised of a control group (C-, *n = 6*) which served as the sham group and underwent anesthesia without receiving irradiation, and an irradiated group (C+, n = 6), which was subjected to a single high-dose of gamma radiation. Both groups received daily administration of saline solution via gavage throughout the entire follow-up period.

The second objective of the study was to evaluate the efficacy of Meloxicam, Pirfenidone, and their combination in mitigating the pathological mechanisms associated with anorectal dysfunction secondary to radiotherapy. Therefore, three treatment groups, were subjected to a single dose of radiotherapy, following the same irradiation protocol as C+ group. Additionally, each group was treated through daily gavage for 28 days, commencing on the day of irradiation: 2 mg/kg Meloxicam (MLX, *n = 6*), 200 mg/kg Pirfenidone (PFD, *n = 6*) and combined therapy with 2 mg/kg Meloxicam and 200 mg/kg Pirfenidone (MLX + PFD, *n = 6*). The dosage regimen was selected in accordance with previous literature studies (Sun et al., 2018; Hofer et al., 2014).

### 2.2 Stereotactic radiotherapy

The radiotherapy procedures were performed under general anaesthesia with intraperitoneal injection with 10 mg/kg Xylazine and 100 mg/kg Ketamine. After anesthesia the animals were positioned in abdominal decubitus, with the legs resting on the sides, onto a headrest, to which they were secured, so that all the animals remained in the same position during the procedures.

### 2.3 Magnetic resonance imaging protocol

To carry out the radiotherapy treatment plan, a prior T1-MRI multi-sliced imaging (MR 5300 Phillips 1.5 T) was performed. Two magnetic resonance imaging (MRI) acquisitions were performed with a total duration of 7 min, using the following settings: *(i)* T1TFE 2D Gradient [*acquisition time = 2* *min*, *TR = 15ms, TE = 5,215* *ms, number of averages = 1, slice thickness = 10mm, slice gap = 120* *mm, slice number = 5, acquisition matrix 308/126 (frequency/phase), in plane phase encoding direction ROW, Flip Angle 20°, ETL = 42*]; *(ii)* T1FFE 3D Gradient [*acquisition time = 5* *min, TR = 25* *ms, TE = 3,761* *ms, number of averages = 1, slice thickness = 1* *mm, slice gap = 0.5* *mm, slice number = 350, acquisition matrix 258/238 (frequency/phase), in plane phase encoding direction COL, Flip Angle 30°, ETL = 1*]. The obtained imaging was loaded into the treatment software, Leksell Gamma Plan (software version 11.3.2), for planning and dose optimization.

### 2.4 Stereotactic radiotherapy planning

**FIGURE 1:**
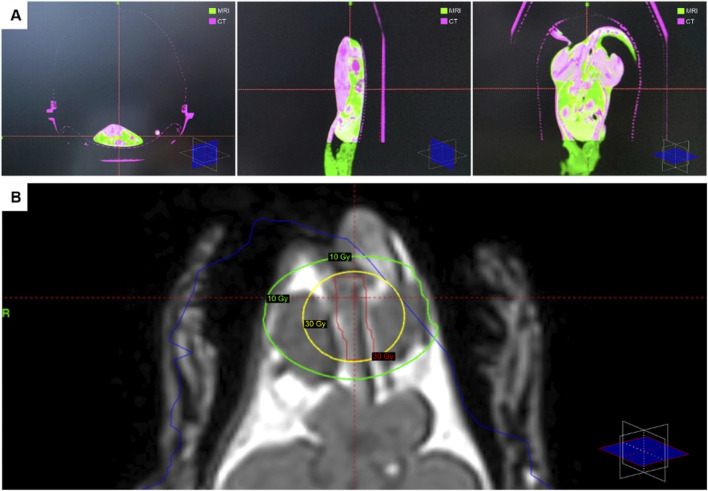
Individually customized stereotactic radiotherapy and dosimetric planning. **(A)** Stereotactic reference imaging in different scanning planes, using co-registered images from MRI and CBCT, automatically overlayed using skeletal landmarks. **(B)** Individually customized treatment plan, highlighting the anal canal (*outlined in red*), the dosimetric prescription (30Gy through the entire organ volume), the 30Gy beam area (*outlined in yellow*) and the surrounding 10Gy isodose (*outlined in green*).

After completing the MRI imaging, the animal holder was transferred to the treatment room of the Leksell Gamma Knife Icon^®^ (Elektra, Sweden). To facilitate the planning and dose optimization processes, a stereotactic reference scan using Cone Beam Computed Tomography (CBCT) was acquired using the integrated Gamma Knife equipment, co-registered with the previously obtained MRI imaging [Figure 1A]. The treatment planning involved contouring the anal canal over a length of 2 cm from the anal edge, including the internal and external anal sphincter in the irradiation area and an automatically optimized dosimetric prescription using Gamma Knife Lightning software (Dubray and Thames, 1994). Furthermore, in the optimization phase the treatment plan goal was to minimize the treatment time and to ensure that the entire target plan receives the minimum prescribed dose, of 30Gy (Hrycushko et al., 2017). The duration of treatment per animal approximated 30 min (15 min for setup and cone-beam computed tomography simulation and 10–15 min for treatment delivery) with isodose levels spanning from 50% to 96%. Each treatment plan was individually customized (through beam sizes, number of shots, and treatment time) using the Gamma Knife Lightning module [Figure 1B].

### 2.5 Anorectal manometry device

**FIGURE 2:**
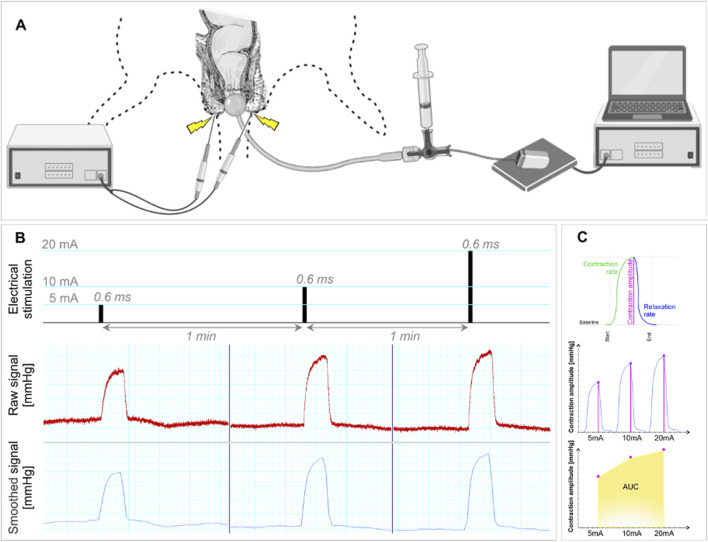
Anorectal manometric recordings demonstrating sphincter contractile response variations. **(A)** Schematic representation of the custom-made device used to perform anorectal manometry. **(B)** Manometric recordings for all three levels of electrical intensities 5mA, 10mA, and 20 mA. **(C)** Contractile responses quantification using Area Under the Curve (AUC).

The anorectal manometry unit was built using commercially available components, enabling precise quantification of radiotherapy-induced sphincter dysfunction [Figure 2A]. Briefly, the device includes an 8Fr Fogarty catheter (Edwards Lifesciences, CA, United States) connected to a 3-way stopcock, a syringe pre-filled with 2 mL of 99% ethanol and a pressure sensor (TRM54PB, Millar Inc., TX, United States) wirelessly connected to a Smartpad TR181 signal acquisition station (Millar, Inc., TX, United States). To position the balloon optimally, we carefully inserted the catheter approximately 3-4 mm from the anal edge, and the balloon was then inflated to a reference pressure, which remained constant throughout the study. The spontaneous contractions of the anal sphincter were used as a guide to ensure the proper placement of the probe.

### 2.6 Electrical stimulation

Preliminary experiments were performed to establish the optimal parameters for *in vivo* evaluation of sphincteric functionality. These tests aimed to determine the electrical stimulation parameters and the application site of the electrical impulses to achieve a firm sphincter contraction. These stimuli were applied without influencing adjacent muscle groups surrounding the anal sphincter, while concurrently addressing potential bias from fatigue due to repeated stimulations. Thus, a series of three transcutaneous electrostimulations were conducted, employing a square wave stimulator system (EXP-ST-CH4, Experimetria Ltd., Hungary), which was interfaced with a multi-control unit (EXP-MCU, Experimetria Ltd., Hungary). Two shielded needle-type monopolar electrodes were positioned at 3 and 9o’clock, effectively encircling the anal edge to target the anal sphincter muscles accurately. The stimulation waveform consisted of rectangular pulses at 10 V, with a pulse-width of 0.6 milliseconds (ms). We applied high-intensity, long duration electrical impulses to directly activate motor axons of the external anal sphincter. This approach aimed to avoid the occurrence of a post-synaptic excitatory response at the spinal cord (H wave) and isolate the muscle’s contractile reaction (M wave) from neural influences, providing an unmediated muscle response to the stimulus while disregarding nerve activity (Gozariu et al., 1998). Furthermore, preliminary tests showed an insufficient contractile response for amplitudes below 5 mA (mA). Respectively, contractions of additional muscle groups or twitching within the hind limbs, were observed for amplitudes exceeding 20 mA, without providing a more efficient sphincteric contractile response. Therefore, to cover the entire interval, three successive pulses with progressively increased intensity were applied: 5, 10, and 20 mA, delivered at 1-min intervals, to ensure optimal data acquisition and prevent muscle fatigue [Figure 2B].

### 2.7 Anorectal manometric evaluation

All animals were subjected to anal sphincter manometric measurements under isoflurane anaesthesia (5% for induction and 2% for maintenance). Manometric recordings were performed at three time points, 1 day prior to radiotherapy, on the 14th and 28th days post-irradiation. After an accommodation period of 5 min, following the anal insertion of the manometry balloon, three successive transcutaneous perianal electrostimulations were applied. All the elicited anal sphincter contractions were recorded and analysed using a PowerLab 16/35 acquisition system (ADInstruments, NSW, Australia). For each contraction, three parameters were quantified: contraction amplitude (*mmHg*), contraction rate and relaxation rate (*mmHg/s*) [Figure 2C].

### 2.8 Histological assays

On the 28th day, after the final manometric recording, the animals were sacrificed under an overdose of isoflurane. A circum-anal incision was then performed to facilitate the *en-bloc* removal of the anus and lower rectum. The entire specimen was fixed in a 10% formalin solution for 24 h, followed by standard tissue processing, paraffin embedding, and serial sectioning at 3 μm thickness. These sections were stained with Hematoxylin & Eosin (H&E) and Masson’s Trichrome for histological analysis. All specimens were scanned using the Aperio AT2 Leica scanner (Leica Biosystems Imaging, CA, United States) for both qualitative and quantitative assessments. Quantification was performed for each animal within its group, and mean values were calculated per group. The first step of the quantitative analysis involved measuring the external anal sphincter thickness, by averaging five measurements taken from five distinct areas. The second step focused on assessing the collagen and muscle tissue ratio in the muscularis propria and the external anal sphincter muscle by an automated computer-assisted morphometry using QuPath v.0.5.1 (Bankhead et al., 2017). Segmentation was performed with K-Means clustering, followed by morphological filtering to remove artifacts. The percentages of collagen and muscular tissue were quantified by calculating the pixel area corresponding to each tissue type relative to the total image area. The collagen and muscular tissue from lamina propria, submucosa, and adventitia were not included in the analysis.

### 2.9 Statistical analysis

For each functional parameter of the anal sphincter (contraction amplitude, contraction rate and relaxation rate), we computed the area under the curve (AUC) based on values obtained during transcutaneous electrostimulation at three different electrical stimulus intensities [Figure 2C]. This approach allowed us to establish a comprehensive quantification of the contractile capacity for each subject across the entire spectrum of electrical stimuli. Subsequently, we calculated the differences in AUC mean (∆AUC ±standard deviation) between measurements taken at 14- and 28-days post-irradiation and those obtained at baseline within each group. For both studies, the assessment of treatment efficacy at each time point and also time-dependent effects between groups was conducted through the application of repeated-measures analysis of variance (ANOVA). Statistical tests were complemented by Šídák’s multiple comparison test for pairwise analysis, which facilitated the evaluation of post-intervention outcomes at each time point between groups.

The statistical significance of the quantitative histological evaluation, including both the thickness of the external anal sphincter and the tissue composition (collagen and muscle percentage) among the five experimental groups, was assessed using ANOVA, followed by Šídák’s *post hoc* test to evaluate differences between treatment effects. All statistical tests were performed using the GraphPad Prism 9.0 software package (GraphPad Software, Massachusetts, USA), with a significance threshold of alpha below 5%.

## 3 Results

### 3.1 Assessment of radiotherapy-induced sphincter dysfunction

#### 3.1.1 Animal welfare

**FIGURE 3:**
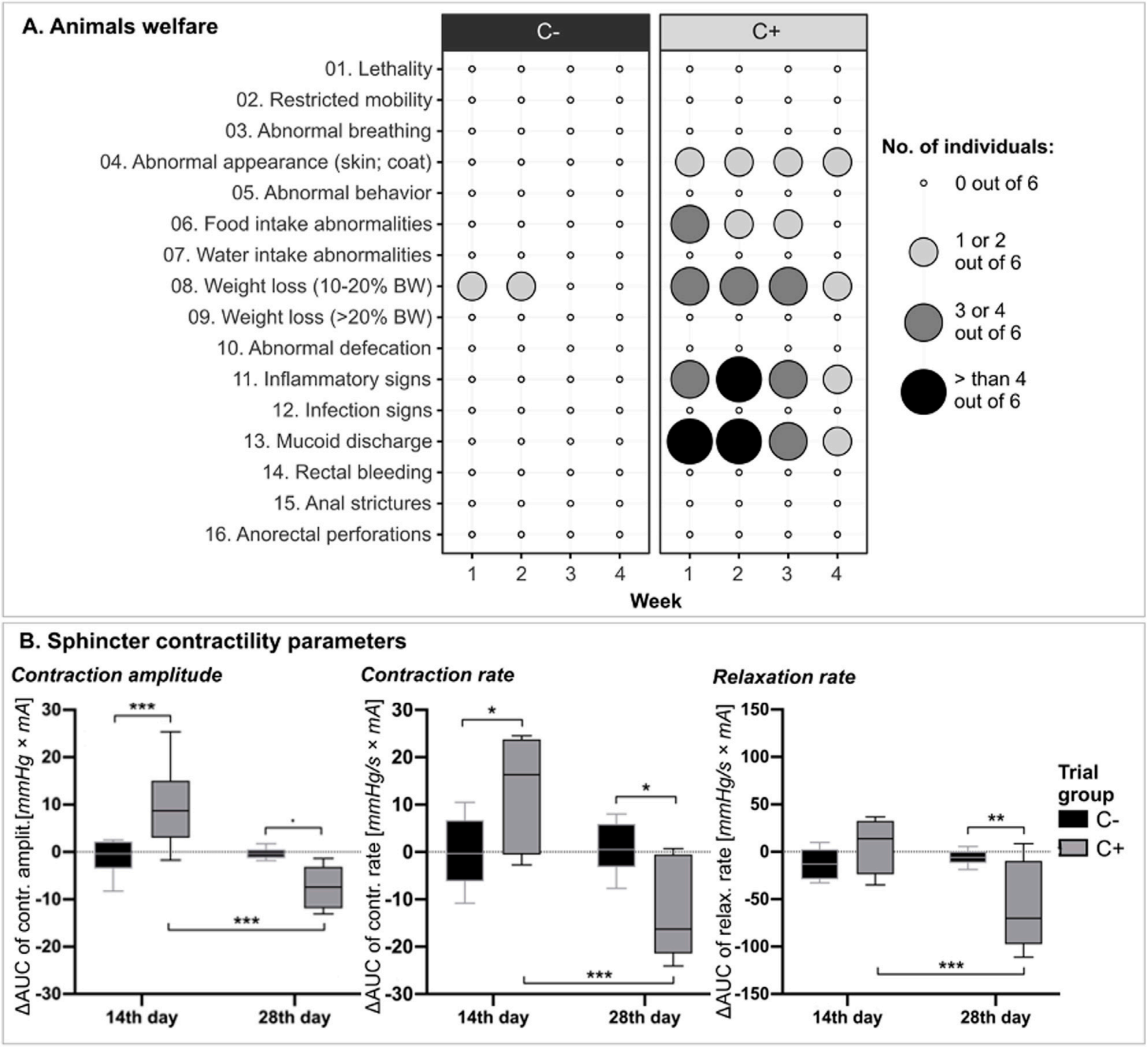
Comparative assessment of animal welfare and sphincter contractility post-radiotherapy. **(A)** Weekly animal welfare monitoring across experimental groups. **(B)** Sphincter contractility parameters represented as ∆AUC ± standard deviation between measurements taken at 14 and 28 days post-irradiation and those obtained at baseline within each experimental group. The differences between groups are shown above the boxplots, while the differences between time points within each group are displayed below the boxplots. Significant differences are represented as * (*p* < 0.05), ** (*p* < 0.01), *** (*p* < 0.001). Additionally, the confidence interval (0.1; 0.05] is indicated by “.”. Abbreviations: C- *(no irradiation, no treatment),* C+ *(30Gy stereotactic radiotherapy, no treatment).*

Throughout the study, animals presented weight loss (<20%) and additionally reduced food intake for the first 3 weeks after irradiation. It is worth noting that one individual from the C- group exhibited weight loss (>10%) during the first 2 weeks, probably due to stress induced by the manometric measurements. However, no additional signs of distress were observed, as quantified by the animal welfare scoring system. Furthermore, all irradiated animals exhibited hyperaemia and discrete oedema in the perianal region, accompanied by increased mucoid secretions, particularly evident for 3 weeks post-irradiation. Conversely, no occurrences of anorectal perforations, anal strictures, bleeding, or wound infections were observed [Figure 3A].

#### 3.1.2 Sphincter contractility

The functional evaluation of the anal sphincter revealed that, within the control group, the contractile parameters exhibited a consistent and unvarying profile across all measurement time points [Figure 3B]. Contraction amplitude was higher in irradiated animals 14 days post-irradiation compared to non-irradiated animals (*p* < 0.001). However, on day 28, the contraction amplitude values in C+ group exhibited a significant decrease compared to the previous timepoint (*p* < 0.001), reaching a lower level than those observed in the control group (*p* < 0.1). Similarly, after 14 days, contraction rates in irradiated animals exhibited higher values than in control group (*p* < 0.05). By day 28, however, these rates significantly decreased, registering values lower than those of the control group (*p* < 0.05) and also below the rates previously observed within the same group (*p* < 0.001). On day 14, there were no differences in sphincter relaxation rate between the C- and C+ groups. Conversely, the relaxation rate recorded in irradiated animals on day 28 displayed a significant decrease compared to control group (*p* < 0.01) and also compared to the prior timepoint within the same group (*p* < 0.001).

#### 3.1.3 Histology

Histological assessment revealed the structural preservation of the anus and anal canal in both groups, although irradiated animals exhibited histoarchitectural alterations at the external anal sphincter (EAS) and the intersphincteric space (ISS). Qualitatively, a reduction in the ISS dimensions was noted, accompanied by an escalation in fibrotic content and adipose cells within group C+, contrasting with negligible alterations in group C- [Figure 5A]. A significant disparity in the thickness of the EAS was observed between groups (C-, 1.17 ± 0.04 mm vs. C+, 0.52 ± 0.13 mm, *p* < 0.01) [Figure 5B]. Futhermore, quantitative analysis of tissue composition indicates a significant increase in collagen deposition in the irradiated group (C+, 34.56% ± 3.8% vs. C-, 18.11% ± 2.5%, *p* < 0.0001) concomitant with a decrease in muscular tissue post-irradiation (C+, 47.06% ± 8% vs. C-, 64.84% ± 3.1%, *p <* 0.0001) [Figure 5C].

### 3.2 Treatment of radiotherapy-induced sphincter dysfunction

#### 3.2.1 Animal welfare

**FIGURE 4:**
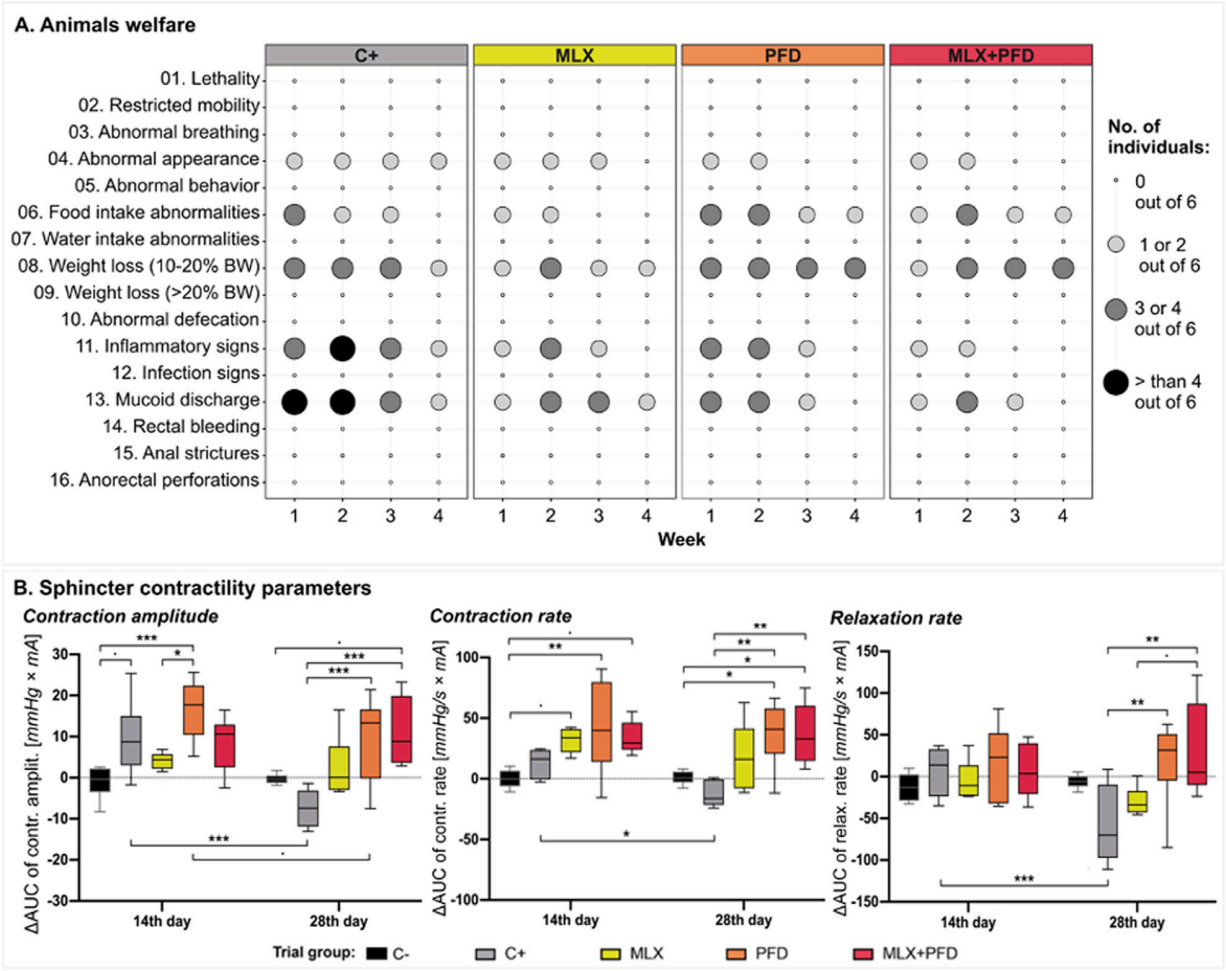
Effectiveness of Pirfenidone and Meloxicam in mitigating sphincter dysfunction post-radiotherapy. **(A)** Weekly animal welfare monitoring across experimental groups. **(B)** Sphincter contractility parameters represented as ∆AUC ± standard deviation between measurements taken at 14 and 28 days post-irradiation and those obtained at baseline within each experimental group. The differences between groups are shown above the boxplots, while the differences between time points within each group are displayed below the boxplots. Significant differences are represented using standard notation: * (*p* < 0.05), ** (*p* < 0.01), *** (*p* < 0.001). Additionally, the confidence interval (0.1; 0.05] is indicated by “.”. Abbreviations: C- *(no irradiation, no treatment),* C+ *(30Gy stereotactic radiotherapy, no treatment),* PFD *(30Gy stereotactic radiotherapy, 200 mg/kg Pirfenidone),* MLX *(30Gy stereotactic radiotherapy, 2 mg/kg Meloxicam),* MLX + PFD *(30Gy stereotactic radiotherapy, 2 mg/kg Meloxicam, 200 mg/kg Pirfenidone).*

Throughout the follow-up period, there were no occurrences of anorectal perforations, anal strictures, bleeding, or wound infections observed across any of the groups. Moreover, all animals maintained normal stool consistency. A slight overall weight loss was noted, with the PFD and MLX + PFD groups experiencing slightly greater weight reduction compared to the MLX or C+ group. Clinical signs of perianal inflammation appeared within each group, at various degrees of severity, without the need for therapeutic intervention or the exclusion of those individuals from the experiment, based on laboratory animal wellbeing scores. In the MLX and respectively MLX + PFD groups, discrete local inflammatory signs were observed, accompanied by mucoid secretions, which, however, improved starting on the 14th day. During the first 2 weeks post-irradiation, 3 individuals from the PFD group presented anal secretions, accompanied by local hyperaemia, with progressive improvement, starting with the 3rd week of follow-up [Figure 4A].

#### 3.2.2 Sphincter contractility

Fourteen days post-irradiation, an elevation in contraction amplitude was noted across all irradiated groups, with a significant difference observed in the PFD group compared to the non-irradiated animals (*p* < 0.001) and respectively to the MLX group (*p* < 0.05). Subsequently, on the last day of follow-up, all groups displayed higher maximum contractile amplitudes compared to their respective baseline, except for the C+ group (*p* < 0.001), which presented significantly reduced values compared to the PFD (*p* < 0.001) and MLX + PFD (*p* < 0.001) groups. There is also a slight, not significant (*p* < 0.1) decrease in contraction amplitude on day 28 in the PFD group compared to the previous measurement. Furthermore, a consistent upward trend of contraction rate was observed after 14 days, which remained elevated above baseline values throughout the entire follow-up period in all treated groups, except the irradiated but untreated group (*p* < 0.05), which exhibited a significantly decreased sphincter contraction rate compared to the PFD (*p* < 0.01) and MLX + PFD (*p* < 0.01) groups at the final manometric measurement. Moreover, an increase in sphincter contraction rate can be observed in the PFD (*p* < 0.05) and MLX + PFD (*p* < 0.05) groups compared to the non-irradiated animals. Sphincter relaxation rate remained relatively constant during the first 14 days in all groups. Surprisingly, on the 28th day of the experiment, both the C+ (*p* < 0.05) and MLX groups demonstrated decreased relaxation rates, indicating a decrease in sphincter relaxation speed compared to baseline values within each group. Additionally, significant differences of this parameter were noted in the PFD and MLX + PFD groups, where improved sphincter relaxation rates were recorded compared to pre-irradiation values and, notably, compared to the values recorded in the C+ group (*p* < 0.01) on the final day of the experiment [Figure 4B]. Notably, for none of the recorded parameters was a statistically significant difference observed between the Pirfenidone-treated group and the group receiving the combined Pirfenidone and Meloxicam therapy.

#### 3.2.3 Histology

**FIGURE 5:**
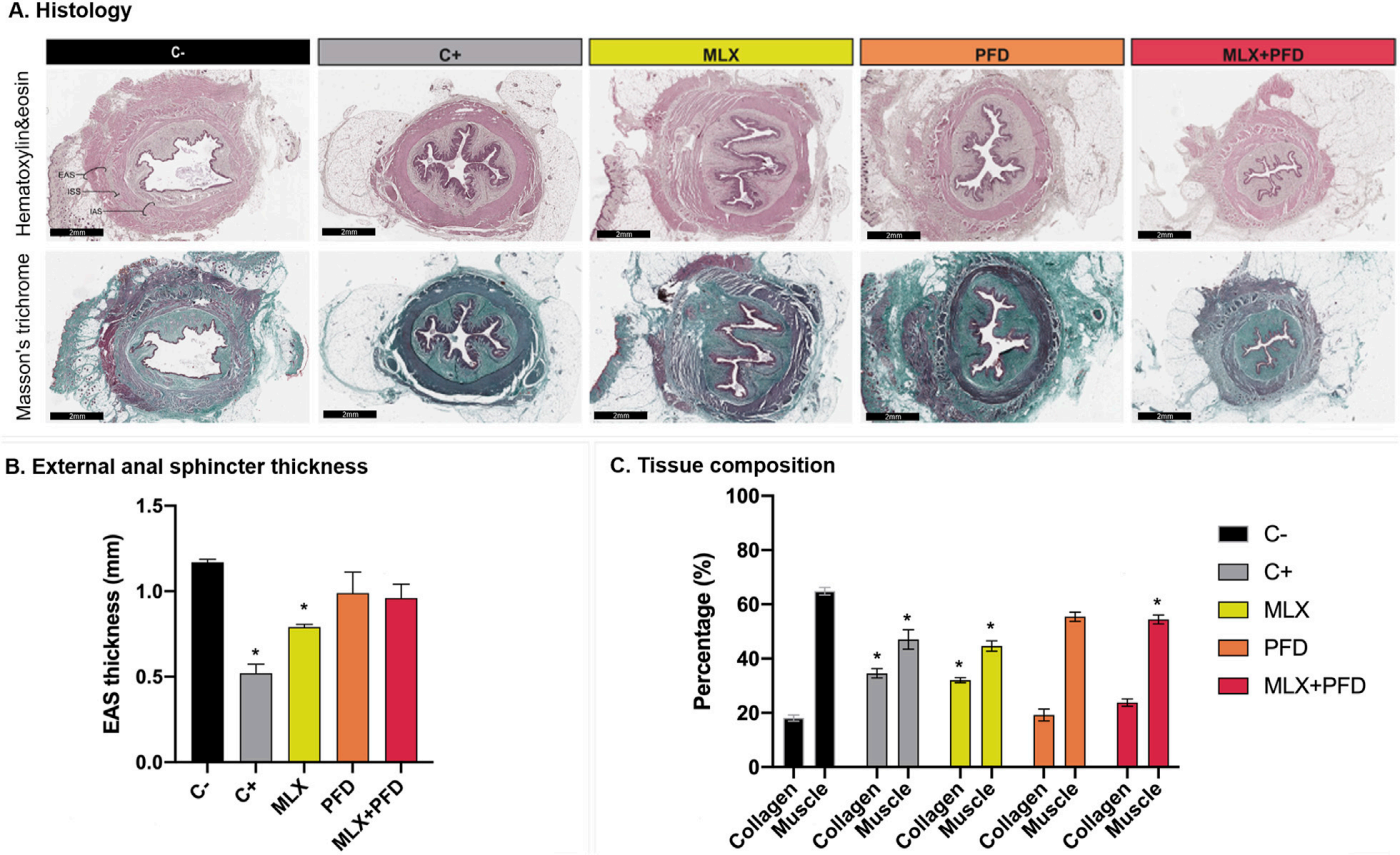
Histopathological evaluation of radiotherapy-induced tissue alterations in the anal sphincter. **(A)** Representative transverse sections of the anal canal at the level of the external anal sphincter, stained with hematoxylin and eosin and Masson’s trichrome, 28 days post-treatment. The scale bar represents 2 mm, with a magnification of ×2. **(B)** Quantitative analysis of external anal sphincter thickness represented as mean ± standard error of the mean across experimental groups. Asterisks (*) indicate statistically significant differences compared to the C- group (*p* < 0.05). **(C)** Tissue composition analysis, showing the percentage of collagen and muscle tissue in each experimental group. Data are presented as mean ± standard error of the mean and asterisks (*) indicate statistically significant differences compared to the C- group (*p* < 0.05). Abbreviations: C- *(no irradiation, no treatment),* C+ *(30Gy stereotactic radiotherapy, no treatment),* PFD *(30Gy stereotactic radiotherapy, 200 mg/kg Pirfenidone),* MLX *(30Gy stereotactic radiotherapy, 2 mg/kg Meloxicam),* MLX + PFD *(30Gy stereotactic radiotherapy, 2 mg/kg Meloxicam, 200 mg/kg Pirfenidone),* IAS *(internal anal sphincter),* EAS *(external anal sphincter),* ISS *(intersphincteric space).*

Substantial alterations in the thickness of the external anal sphincter and histoarchitectural modifications of the intersphincteric space were noted, with preservation of the histological integrity of the anus and anal canal across all groups. Qualitative analysis of the ISS revealed fibromatous alterations with a concurrent rise in the relative number of adipose cells in the C+ group, mild congestion associated with increased vascular density in the MLX group, and an elevation of the adipocytes, devoid of other noteworthy changes, in the PFD and MLX + PFD groups, respectively [Figure 5A]. Quantitative analysis of tissue sections revealed a significant reduction in external anal sphincter thickness in the MLX group, whereas only a slight decrease was observed in the PFD and MLX + PFD groups compared to the non-irradiated controls (MLX, 0.79 ± 0.04 mm, *p* < 0.01; PFD, 0.99 ± 0.3 mm, *p* > 0.05; MLX + PFD, 0.96 ± 0.2 mm, *p* > 0.05) [Figure 5B]. Furthermore, tissue composition assessment revealed that the MLX group exhibited a significant increase in collagen content compared to the non-irradiated animals (MLX, 32.04% ± 1.9%, *p* < 0.0001), while muscular tissue was also reduced (MLX, 44.63% ± 4.3%, *p <* 0.0001). The combined treatment (MLX + PFD) resulted in a slight increase in collagen content (23.76% ± 2.9%, *p* > 0.05), accompanied by a significant decrease in muscle tissue compared to the non-irradiated group (MLX + PFD, 54.41% ± 3.5%, *p* < 0.05). Notably, in the group treated with Pirfenidone, both collagen (PFD, 19.23% ± 4.8%, *p >* 0.05) and muscle content (PFD, 55.43% ± 3.7%, *p* > 0.05) did not significantly differ from the control group, suggesting an antifibrotic effect as well as a protective role in maintaining the structural integrity of the muscularis propria [Figure 5C].

## 4 Discussion

In light of significant advancements in the treatment of solid tumors, the overall survival rates of cancer patients have notably improved, leading to a rapid increase in the population of cancer survivors. This has shifted the research emphasis towards mitigating the side effects of cancer treatments that adversely impact patients’ quality of life. A foundational step in addressing this challenge involves the development of reliable animal models for evaluating the efficacy and safety of novel therapeutics. This study aimed to create a specific animal model to study radiotherapy-induced anal sphincter dysfunction, utilizing gamma knife technology to deliver ionizing radiation precisely to the anal canal and external and internal anal sphincter, minimizing the impact on adjacent structures. Noteworthy is the absence of similar models in existing scientific literature that specifically investigate SBRT targeting the rectum and perianal region in rat models. Employing a cobalt-60 source, SBRT delivers high-dose radiation precisely to tumour locations while minimizing damage to surrounding tissues, closely mirroring clinical practices aimed at sparing organs at risk (Vaniqui et al., 2020). In clinical settings, radiotherapy is typically administered using a fractionated dosing regimen, where the total dose is divided into multiple smaller fractions to mitigate toxicity and preserve normal tissue function. The fractionation strategy leverages the differential radiosensitivity of tumor and normal tissues, as described by the linear-quadratic model, where the α/β ratio plays a crucial role in determining the response to radiation exposure. For rectal tissue, the α/β ratio is estimated to be approximately 3–5 Gy, classifying it as a late-responding tissue that is particularly prone to long-term fibrosis following radiation therapy (Dubray and Thames, 1994). However, in this study, we deliberately employed a single high dose of 30 Gy to ensure the induction of a robust inflammatory and fibrotic response, allowing for a reliable model to assess therapeutic interventions aimed at mitigating radiation-induced anorectal dysfunction. The rationale for selecting a single high dose stems from existing preclinical studies demonstrating that doses exceeding 24 Gy are sufficient to induce severe rectal toxicity, including obstruction and fibrosis (Hrycushko et al., 2017). This approach aligns with prior research investigating the impact of high-dose irradiation on rectal tissue, wherein significant histopathological changes and functional impairments were observed following exposure to similar radiation doses (van den Aardweg et al., 2003).

The simplicity and efficiency of the single-session delivery system of the Gamma Knife render it exceptionally suited for screening a large number of potentially effective drugs in a time-efficient manner (Song et al., 2021). Our investigation emphasized achieving reproducible results in terms of tissue damage and sphincter dysfunction, addressing both external and internal anal sphincter challenges. The observed local changes post-SBRT, including hyperaemia, oedema in the perianal region, increased mucoid secretions, and impaired sphincter functionality, corroborate with existing findings on rectal radiation injuries (O'Brien, 2001), highlighting the model’s relevance and potential utility in refining therapeutic approaches. By inducing a controlled fibrotic response, this model facilitates the assessment of antifibrotic agents in preserving anal sphincter integrity and function, with the goal of identifying potential clinical applications. Future investigations should explore whether fractionation alters the severity of fibrosis and whether the tested pharmacological agents maintain their efficacy under different fractionation regimens. The insights gained from this model may facilitate the translation of novel therapeutic strategies into clinical practice, ultimately improving patient outcomes following pelvic irradiation.

Furthermore, in the present paper we report the successful development of an innovative anorectal manometer for rats, capable of detailed evaluation of compromised sphincter contractility. Anorectal sphincter dysfunction, manifesting around 4 weeks post-radiotherapy (Yeoh et al., 1998), has been associated with an increased risk of developing late gastrointestinal toxicities (Choi et al., 2016). This underscores the utility of early anorectal manometry assessment as an essential method for quantifying radiation-induced toxic effects. Thus, our device capabilities in recording the anal sphincter contractility as a contraction slope, offers a comprehensive evaluation beyond the mere measurement of anal contractions and resting pressure. Therefore, our model emerges as a valuable asset for exploring therapeutic strategies aimed at alleviating the detrimental consequences of radiotherapy on anorectal function.

Previous studies underline the existence of a complex interaction between radiation exposure and anal sphincter performance. Birnbaum et al. (1992) observed minimal immediate effects on the anal sphincter from preoperative radiation therapy, noting a significant increase in minimal sensory threshold but no substantial change in mean maximal squeeze or resting pressures. Iwamoto et al. (1997) expanded on these findings, showing that radiotherapy led to a significant increase in anal canal resting pressure and a notable decrease in rectal compliance and maximum tolerable volume, with these effects developing during radiotherapy and progressing over time. Gervaz et al. (2001) further quantified the impact of chemoradiation on anal sphincter function, demonstrating a significant decrease in resting pressures post-chemoradiotherapy and highlighting the importance of adequate shielding of the anal sphincter during treatment for low rectal cancers. These studies suggest that the initial increase in contractile parameters post-radiotherapy that was observed in day 14 of our study may be attributed to compensatory mechanisms or inflammatory responses, leading to heightened anal sphincter activity. Over time, however, radiation-induced damage to the sphincter’s structural integrity, nerve supply, and surrounding tissues progresses, resulting in decreased sphincter function and late anorectal dysfunction. Our results align with the observed biphasic effect of radiotherapy on anal sphincter contractility, demonstrating an initial increase in contractile parameters followed by a decrease below baseline levels, reflecting a progression from an acute phase to a chronic phase of deterioration and dysfunction. Furthermore, our study delineates a divergence from prior research (Yeoh et al., 2000), which indicated no discernible reduction in the thickness of the external anal sphincter post-radiotherapy. In contrast to these earlier assertions, our investigation unveiled a significant diminution in EAS thickness following radiotherapy exposure, heralding a more profound impact on sphincteric function than has been previously recognized. This morphological alteration may provide a tangible substrate for the functional changes we observed, establishing a direct link between structural modifications of the EAS and subsequent anal sphincter dysfunction.

The detrimental impact of ionizing radiation on anorectal function is largely attributed to its fibrogenic effects, which manifest as abnormal collagen deposition and heightened fibroblast activation within the anal sphincter (Wang et al., 2020). These processes are driven by specific molecular pathways, notably TGF-β1/Smad and p38 MAPK, leading to fibrosis and the ensuing anorectal dysfunction (Vallée et al., 2017). Additionally, radiation prompts direct DNA damage, generating reactive oxygen species (ROS) that contribute to a broad spectrum of pro-inflammatory effects (Ahamed and Laurence, 2017). In addressing these mechanisms, the present study evaluated the efficacy of Meloxicam, Pirfenidone and their combination in counteracting radiation-induced anal dysfunction.

Pirfenidone exerts its anti-inflammatory, antioxidative, and antifibrotic effects through multiple molecular pathways. It modulates the TGF-β1/Smad3 signaling cascade, reducing fibroblast proliferation and collagen synthesis, which are key processes in radiation-induced fibrosis. Additionally, Pirfenidone inhibits the p38 MAPK pathway, which plays a central role in fibroblast activation and extracellular matrix (ECM) deposition (Sartiani et al., 2022; Lv et al., 2020). Beyond TGF-β1 inhibition, Pirfenidone also suppresses the NF-κB pathway, thereby downregulating pro-inflammatory cytokines such as TNF-α, IL-1β, and IL-6, which contribute to radiation-induced tissue damage and chronic inflammation (Wang et al., 2020). Moreover, Pirfenidone exerts antioxidant properties by attenuating reactive oxygen species production, thereby reducing oxidative stress-induced fibroblast activation and epithelial-mesenchymal transition (EMT), two major drivers of post-radiotherapy fibrosis. This effect is partly mediated through its ability to downregulate NADPH oxidase activity, a key enzymatic source of ROS (Simone et al., 2007). Furthermore, Pirfenidone has been shown to modulate the PI3K/Akt/mTOR pathway, which is involved in fibroblast proliferation, apoptosis resistance, and tissue remodeling following injury (Vallée et al., 2017). In addition to these pathways, recent evidence highlights that Pirfenidone also inhibits the Hedgehog (Hh) signaling pathway, which plays a pivotal role in fibrotic progression (Didiasova et al., 2017). Normally inactive in adult tissues, Hedgehog signaling can be aberrantly reactivated following tissue injury, promoting myofibroblast differentiation, ECM accumulation, and excessive collagen deposition. In the context of radiation-induced fibrosis, activation of the Hedgehog pathway has been associated with increased TGF-β expression, connective tissue growth factor (CTGF) upregulation, and persistent fibroblast activation, leading to progressive tissue stiffening and dysfunction (Wang et al., 2013). By interfering with Hedgehog ligand-dependent signaling, Pirfenidone effectively reduces fibroblast-to-myofibroblast transition, mitigates collagen overproduction, and attenuates pathological ECM remodeling, further reinforcing its antifibrotic potential (Didiasova et al., 2017; Prasse et al., 2019).

By modulating these interconnected mechanisms, Pirfenidone attenuates both early inflammatory responses and long-term fibrotic remodeling, thereby preserving the structural integrity of the anal sphincter and maintaining contractile function post-radiotherapy (Latella and Viscido, 2020). Its antifibrotic and anti-inflammatory efficacy has been demonstrated in various preclinical and clinical models, including pulmonary, renal, hepatic, and intestinal fibrosis (Vallée et al., 2017; Ma et al., 2018). Beyond its approved use for idiopathic pulmonary fibrosis, Pirfenidone has shown promising effects in mitigating radiation-induced lung injury (Chen et al., 2022) and is currently under investigation in clinical trials for preventing such damage. Its ability to reversibly inhibit fibroblast activation, suppress collagen deposition, and modulate inflammatory cytokine production further underscores its broad applicability in counteracting radiation-induced tissue damage (Qin et al., 2018). These multifaceted molecular actions establish a strong mechanistic foundation for its therapeutic role in anal sphincter dysfunction secondary to radiotherapy, bridging the gap between pathophysiology and targeted interventions. Ultimately, these findings support its clinical translation, reinforcing its significance in managing post-radiotherapy anorectal dysfunction.

Meloxicam, the second agent investigated in our study, acts as a non-steroidal anti-inflammatory drug by inhibiting cyclooxygenase (COX) enzymes, primarily COX-2. Research underscores NSAIDs’ capacity to protect normal tissues from radiation-induced damage through mechanisms such as increasing arachidonic acid concentrations, enhancing cellular superoxide dismutase levels, and modulating cytokine expression (Lee and Stupans, 2002). By blocking prostaglandin synthesis, key players in inflammation, NSAIDs can mitigate the initial inflammatory response elicited by radiation (Milas, 2003). Several NSAIDs, including aspirin, ibuprofen, and sulfasalazine, have undergone investigation in both preclinical and clinical realms with varying results (Northway et al., 1988). However, Meloxicam and similar COX-2 inhibitors exhibit distinctive advantages, showing a positive effect on the survival of gamma-irradiated mice subjected to total body irradiation (Hofer et al., 2014) and presenting significant radiosensitizing effects to augment treatment outcomes (Laube et al., 2016). In clinical settings, COX-2 inhibitors, such as celecoxib, have successfully reduced the acute side effects of radiotherapy and the need for analgesics, demonstrating beneficial effects (Feigenberg et al., 2003). Yet, the specific impact of Meloxicam on radiation-induced anal dysfunction remains under-explored, indicating a compelling direction for future research. Notably, the MLX group showed less improvement compared to the PFD and combination treatment groups, indicating that NSAID monotherapy may not fully counteract radiation-induced fibrosis and secondary anal sphincter dysfunction. This limitation likely arises from NSAIDs’ focus on the acute inflammatory phase, without adequately addressing the fibrotic pathways crucial for preventing structural changes that lead to dysfunction.

This study demonstrated that both Pirfenidone monotherapy and combination therapy with Meloxicam improved sphincter contractility 28 days post-radiotherapy, exceeding baseline values. However, the combination therapy did not provide an additive benefit, as its functional effects were comparable to those of Pirfenidone alone. These findings indicate that Meloxicam did not enhance the therapeutic efficacy of Pirfenidone in improving sphincter function. From a histological perspective, quantitative tissue analysis confirmed that Pirfenidone monotherapy exhibited the most pronounced protective effects, significantly reducing collagen deposition and preserving muscular tissue. In contrast, these benefits were less evident in the Meloxicam-treated and combination therapy groups, suggesting that Pirfenidone exerts its effects predominantly through direct antifibrotic and myoprotective mechanisms, independent of the addition of Meloxicam. While the combination therapy successfully mitigated collagen deposition, its myoprotective effect was less pronounced, possibly due to the inhibition of COX-2 by Meloxicam, which may have interfered with muscle regeneration and the activation of satellite cells, essential for tissue repair. Regarding external anal sphincter thickness, both Pirfenidone monotherapy and combination therapy effectively counteracted its reduction, reinforcing their potential as targeted therapeutic strategies for preserving anorectal function following radiotherapy. However, since combination therapy did not confer additional advantages over Pirfenidone alone, these results suggest that Meloxicam’s anti-inflammatory properties do not synergize with Pirfenidone’s antifibrotic and myoprotective mechanisms in this context.

These findings position Pirfenidone as the most effective approach for maintaining sphincter function and structural integrity post-radiotherapy. While combination therapy mitigated fibrosis, it did not enhance muscle preservation, highlighting the need for further investigations into the interplay between anti-inflammatory and antifibrotic treatments in radiation-induced tissue damage.

Our study acknowledges certain limitations, primarily the brief post-radiotherapy follow-up period, concluding on day 28. This duration was chosen deliberately to validate the proposed animal model for anorectal dysfunction secondary to radiotherapy, and concurrently assess the therapeutic efficacy within the same timeframe. Furthermore, the mechanisms underlying the effectiveness of Pirfenidone and Meloxicam have not been extensively studied, necessitating further research. Despite these limitations, our study not only advances our understanding of the key pathogenic mechanisms responsible for anal sphincter dysfunction in oncological patients undergoing adjuvant pelvic radiotherapy but also introduces a novel animal model in preclinical research. This model represents a step forward in bridging the gap between preclinical discoveries and clinical applications, specifically aimed at enhancing treatments for anal sphincter dysfunction in cancer patients following pelvic radiotherapy.

## 5 Conclusion

The exploration of protective measures against the impact of radiation therapy on anal sphincter function underlines the critical need for strategies to mitigate anal dysfunction among oncology patients receiving pelvic radiation therapy. This underscores the importance of early detection and potential intervention strategies to mitigate late gastrointestinal toxicity, reinforcing the necessity for further research in this area to improve clinical outcomes. Integrating our findings, we ascertain that Pirfenidone is effective both as a single-drug treatment and in combination with Meloxicam, not only in enhancing functional manometric outcomes but also in the structural preservation of the anal sphincter, heralding a novel paradigm in the management of radiation-induced anal sphincter dysfunction. However, our results demonstrate that the addition of Meloxicam does not enhance the therapeutic efficacy of Pirfenidone, as the combination therapy did not provide an additive benefit in either functional recovery or structural preservation. Future research should focus on deciphering the molecular pathways driving radiation-induced fibrosis, including the interplay between inflammatory mediators, extracellular matrix remodeling, and myogenic repair mechanisms. Additionally, exploring novel pharmacological agents and combination regimens targeting both fibrotic progression and muscle regeneration could pave the way for more effective therapeutic interventions. These advancements are crucial to refining personalized treatment approaches, ultimately improving functional recovery and quality of life in post-radiotherapy patients.

## Data Availability

The raw data supporting the conclusions of this article will be made available by the authors, without undue reservation.
